# Heart Rate Variability and Performance of Commercial Airline Pilots during Flight Simulations

**DOI:** 10.3390/ijerph16020237

**Published:** 2019-01-16

**Authors:** Xiaodong Cao, Piers MacNaughton, Leslie R. Cadet, Jose Guillermo Cedeno-Laurent, Skye Flanigan, Jose Vallarino, Deborah Donnelly-McLay, David C. Christiani, John D. Spengler, Joseph G. Allen

**Affiliations:** 1 Department of Environmental Health, Harvard T.H. Chan School of Public Health, Boston, MA 02215, USA; xcao@hsph.harvard.edu (X.C.); piers.macnaughton@gmail.com (P.M.); lcadet@hsph.harvard.edu (L.R.C.); memocedeno@mail.harvard.edu (J.G.C.-L.); flanigan@hsph.harvard.edu (S.F.); jvallari@hsph.harvard.edu (J.V.); ddonnellymclay@hsph.harvard.edu (D.D.-M.); dchris@hsph.harvard.edu (D.C.C.); spengler@hsph.harvard.edu (J.D.S.); 2 Department of Medicine, Massachusetts General Hospital/Harvard Medical School, Boston, MA 02114, USA

**Keywords:** heart rate variability, pilot, carbon dioxide, stress, flight maneuver

## Abstract

Pilots undergo a variety of stressors that may affect their performance during all phases of flight. Heart rate variability (HRV) has been considered as a reliable indicator of the parasympathetic and sympathetic activities of human autonomic nervous system, which can be used to characterize the sympathetic stress response of pilots during flight. In this study, thirty active commercial airline pilots were recruited to fly three flight segments in a Federal Aviation Administration (FAA)-certified A320 flight simulator with each segment at a different carbon dioxide (CO_2_) concentration on the flight deck. The pilots performed a series of maneuvers of varying difficulty, and their performance was evaluated by FAA designated pilot examiners. The HRV metrics (SDNN, RMSSD and LF/HF ratio) of each pilot both before and during flight simulations were measured with a Movisens EcgMove3 sensor. The average SDNN, RMSSD and LF/HF ratio of the pilots during flight simulations were 34.1 ± 12.7 ms, 23.8 ± 10.2 ms and 5.7 ± 2.8 respectively. Decreased HRV was associated with aging, obesity and performing difficult maneuvers. Both CO_2_ exposure and HRV had an independent effect on the pilot performance, while their interaction was not significant. The generalized additive mixed effect model results showed that a pilot performed better on a maneuver when his stress response was lower, as indicated by higher SDNN and RMSSD and lower LF/HF ratio. An interquartile range (IQR) increase in SDNN (21.97 ms) and RMSSD (16.00 ms) and an IQR decrease in LF/HF ratio (4.69) was associated with an increase in the odds of passing a maneuver by 37%, 22% and 20%, respectively.

## 1. Introduction

The International Air Transport Association (IATA) expects 7.2 billion passengers to travel by air in 2035, a near doubling of the 3.8 billion passengers that travelled in 2016 [1]. Within the U.S. Federal Aviation Administration’s (FAA) Air Traffic Control Organization alone, over 2.5 million airline passengers travel on 43,000 airline flights every day [2]. Billions of passengers travel by air every year; however, compared to other forms of transportation, flying is the safest, with only 138 onboard and one external fatality worldwide in 2016 [3].

As of 2017, there were an estimated total of 609,306 pilots within the FAA’s jurisdiction, including 159,825 airline transport pilots [4]. Before an individual can become a pilot, they must obtain a medical certificate, which indicates that they are healthy enough to operate an airplane. Medication usage, medical history, use of corrective lenses, surgeries, and recent visits to health care professionals are all items that must be disclosed to the Aviation Medical Examiner (AME) by the applicant. Though having strict requirements of qualification, the actual performance and mental health of airplane pilots on the flight deck still warrants further intervention. An anonymous survey-based study showed that some pilots may suffer from depressive symptoms that they do not disclose to the AME or their primary care manager due to the fear of negative career impacts [5]. Pilots’ attempts to protect their careers through non-disclosure of their symptoms prevents them from receiving proper treatment. Airline pilots also reported heightened self-rated fatigue and irregular sleep during international flights [6,7].

Pilots undergo a variety of physical, psychological, and physiological stressors that affects their performance during the flight. Examples include flight deck humidity, family illnesses or death, or fatigue and physical deconditioning. To ensure flight safety, it is necessary to have a deeper insight into the stress levels of pilots during flight, and how stress impacts their performance. The occupational stress and workload can be estimated through physiological indicators such as cortisol levels in saliva, respiration rate and heart rate variability (HRV) [8,9,10]. A recent study [11] indicated that the stress of pilots was elevated as indicated by lower HRV, when switching from analog to digital visual presentations of the flight and navigation data.

The stress response system is comprised of the autonomic nervous system (ANS) and the hypothalamic-pituitary-adrenal (HPA) axis [12]. Activation of the sympathetic nervous system (SNS) with inhibition of the parasympathetic nervous system (PNS) triggers the acute response to both physical and psychological stress, also known as the fight-or-flight response [13]. During the stress response, the HPA axis is initiated by the release of the corticotrophin-releasing hormone from the hypothalamus, which results in a series of endocrine changes that culminates with the release of cortisol from the adrenal cortex [14]. The PNS plays an integral role in alleviating the stress response of individuals by inhibiting the SNS and HPA axis [12,15]. The PNS also regulates the “rest and digest” functions that calm the body down and dampen the stress response [15,16]. HRV is a measure of the variability in the length of time between heart beats, which serves as a proxy for the dynamic interplay between the parasympathetic and sympathetic branches of the ANS [17]. Current neurobiological evidence suggests that HRV indices can be used as an objective physiological indicator of stress [18]. HRV can be measured in both a time-domain and a frequency-domain [19,20]. Time-domain HRV indices represent the variability in the time intervals between successive heartbeats. SDNN (standard deviation of the normal to normal interval) and RMSSD (root mean square of successive differences between normal heartbeats) are the two most commonly-used HRV time-domain indices. SDNN reflects the total heart rate variability correlated with ANS activities, while RMSSD is more of a marker of parasympathetic regulation of heart. Both higher SDNN and RMSSD have been associated with physiological resilience against stress [18,21]; low variability could be attributed to pathologies such as hypertension, diabetes, and depression, all of which are associated with stress and decreased cognitive function [22,23].

Frequency-domain measurements describe the power distribution of HRV as a function of frequency. The low frequency (LF) component (0.04 to 0.15 Hz) of HRV is produced by both SNS and PNS activities. An increased LF power may reflect increased sympathetic activity during mental stress and exercise [20]. The high frequency (HF) component (0.15 to 0.4 Hz) of HRV is primarily produced by PNS activity and highly correlated with the RMSSD time-domain measures [24]. Lower HF power is correlated with higher stress, panic, anxiety or worry [19]; therefore, the ratio of LF power to HF power (LF/HF ratio) can be used to estimate the balance between SNS and PNS activity [23]. A low LF/HF ratio reflects the dominance of PNS activity, when people conserve energy and engage in tend-and-befriend behaviors. Conversely, a high LF/HF ratio indicates sympathetic dominance, which occurs when people engage in fight-or-flight behaviors or parasympathetic withdrawal.

The performance of pilots may also be affected by environmental conditions on the flight deck such as temperature, aircraft vibration, noise, air quality and ventilation. The flight deck has been under studied, however; nearly all of the research to date on these environmental factors in airplanes, has focused on conditions in the airplane cabin [25,26,27,28,29]. Specific to air quality, a focus of our current study, one study of 179 U.S. domestic flights, Cao et al. [30] found an average CO_2_ concentration of 1353 ± 290 ppm (mean ± SD) during all flight phases, but as high as nearly 3000 ppm during boarding, a time when the flight deck door is usually open. The equivalent outside air ventilation rates could only meet the minimum value of 4.7 L/s/p as required by Federal Aviation Regulations [31] 42% of time during boarding and 73% of time during flying. Data for conditions on the flight deck are more limited. The European Aviation Safety Agency (EASA) measured the CO_2_ concentrations in the cockpits of eight B787 airplanes and 61 other types of airplanes [32]. The mean CO_2_ concentration on the B787 flight deck was 603 ppm with a range of 473 to 1229 ppm. On the flight deck of other airplanes, the mean CO_2_ concentrations were 835 ppm (629–1918 ppm) and 753 ppm (594–1976 ppm) for short-haul and long-haul flights, respectively.

Exposure to CO_2_ at these levels has been shown to be associated with detrimental effects on cognitive function and increasing prevalence of health symptoms in other indoor settings [33,34,35,36]. Further, our recent study that focused on CO_2_ and airplane pilots [37] demonstrated that CO_2_ concentrations impact airline pilot performance at levels occasionally observed on the flight deck. Compared to segments at a CO_2_ concentration of 2500 ppm, the odds of passing a maneuver in flight simulations were 1.52 (95% CI: 1.02–2.25) times higher when pilots were exposed to 1500 ppm and 1.69 (95% CI: 1.11–2.55) times higher when exposed to 700 ppm [37]. Based on prior studies showing the potential for elevated CO_2_ in the airplane and an impact of CO_2_ on pilot performance, the aims of our present study were to: further investigate the stress response of pilots when conducting flight maneuvers of varying difficulty at different CO_2_ concentrations during the flight simulations; and to evaluate how sympathetic stress response, as indicated by HRV metrics impact, the performance of pilots. Using a crossover repeated measures study design, we recruited thirty active commercial airline pilots and had them complete a series of three simulated flights in an FAA-certified A320 flight simulator at three CO_2_ conditions: 700 ppm, 1500 ppm, and 2500 ppm. Pilots had HRV monitored for the duration of the flight, and the flight performance of pilots was rated by FAA designated pilot examiners. We sought to examine the effects of different influencing factors on pilots’ HRV, and in turn the effect of HRV on the flight performance.

## 2. Materials and Methods

### 2.1. Participants

Thirty active commercial airline pilots participated in this panel study in March-May, 2017. All participants were currently qualified to fly the Airbus A320 aircraft. Their demographic and flight experience information are presented in Table 1.

All recruited pilots were male, which fits the current demographic of airline pilots globally (94% male) [38]. Pilots were paired to create fifteen teams, and each flight team completed three simulated flight tests in an FAA-certified A320 flight simulator. During the approximately three-hour long simulation, each pilot was the pilot flying for half of the simulation, and the pilot monitoring for the other half. The pilot who flew first for the first simulation flew first for the remaining two sessions, and pilots sat in the left or right seat based on where they typically sat during actual flight. The Institutional Review Board (IRB) of the Harvard T.H. Chan School of Public Health reviewed and approved the study protocol. Informed consent was obtained from all participants.

### 2.2. Experimentation

**Figure 1:**
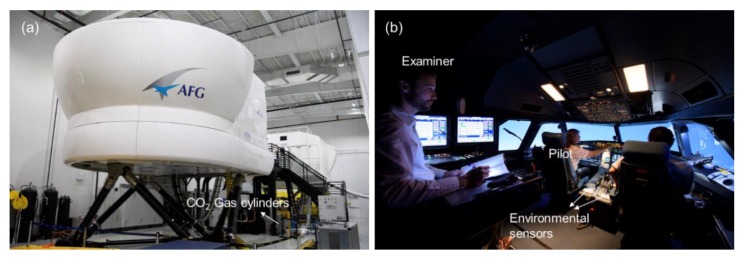
(**a**) An FAA-approved A320 flight simulator; (**b**) Flight simulation test.

An FAA-approved A320 flight simulator (AFG, Inc., Fort Lauderdale, FL, USA) for pilot training and certification was used in the study (Figure 1). A series of flight maneuvers of varying difficulty (A, B and C), grouped into three sequences, were programmed into the flight simulator. The descriptions for each flight maneuver are summarized in the Table S1 (see supplemental material). The detailed FAA definition of each of the maneuver and rating criteria can be found in [39,40]. There were also transitional periods between adjacent maneuvers, which were we defined collectively as ‘Gap’ in our analysis so we could delineate time periods when pilots were actually performing maneuvers. Each pilot on each flight team performed all of the maneuvers during each of the three simulated flights. The three flight simulation tests were executed in different order based on the airport that the pilots departed from—Boston Logan International, New York LaGuardia Airport/New York Kennedy Airport, and Ronald Reagan Washington National Airport.

Each pilot team flew one flight at each of the three targeted CO_2_ conditions: 700 ppm, 1500 ppm, 2500 ppm. Prior to each session, the CO_2_ level was adjusted back to a background concentration of 400 ppm with outdoor air. The CO_2_ concentration was modified by introducing ultra-pure CO_2_ (99.9% pure CO_2_, 0.1% H_2_O) from a gas cylinder into the simulator through the simulator’s ventilation system. Two environmental sensors (HOBO model MX1102, Onset Computer Corporation, Bourne, MA, USA) were installed on the left and right sides of the center console between the pilot seats to monitor real-time CO_2_ concentrations. The other environmental conditions were held constant during the flight simulation tests: total ventilation rate (850 L/s), temperature (24 ± 1 °C) and relative humidity (47 ± 2%). Pilots and examiners were both blinded to test conditions and the order of exposures was randomized. More specifics on the experimental methodology can be found in Allen et al. [37].

Three FAA designated examiners participated in the evaluation of pilot performance during the simulated flights, with the majority of ratings performed by one examiner (Examiner 1: 65% of flights, Examiner 2: 24% of flights; Examiner 3: 11% of flights). During each simulation, one of the three examiners was seated at the control console located behind the pilots. The examiner had full control over the simulator and an elevated vantage point to observe the pilot actions and communications. The pilot flying and the pilot monitoring worked as team with different responsibilities during each maneuver; the rating by the examiner was an indication of their combined flight performance. The examiners rated each maneuver according to standardized protocols used by the FAA during flight certification tests. The overall passing rates of pilots on each maneuver are presented in Figure S1.

The Movisens EcgMove3 sensor (Movisens, GmbH, Karlsruhe, Germany) [41], which was worn on a chest belt underneath the clothes with direct skin contact, was used to measure the electrocardiogram (ECG) data of each pilot from 10 min before entering the flight simulator to the end of simulations. The sensor collected single channel ECG data with a resolution of 12 bits and a sampling rate of 1024 Hz. The Movisens software was used to convert the ECG signals into time-serial HRV indices: SDNN (ms), RMSSD (ms), LF power (ms^2^), HF power (ms^2^) and LF/HF ratio. The HRV indices were calculated in an interval of 30 s (the minimum interval of EcgMove3 sensor).

### 2.3. Data Analysis

Multivariate linear models were used to test the fixed effect estimates of potential influencing factors on HRV indices for each maneuver. The influencing factors tested include age, body mass index (BMI), regular flight experience as a pilot or in simulation, CO_2_ condition settings and difficulty level of the maneuver, controlling for flight profile number (i.e., order of maneuvers) and examiner. Generalized additive mixed effect models (GAMM) were used to test the relationships between HRV indices and pilot performance, controlling for CO_2_ condition, maneuver difficulty, flight profile number, and examiner, and treating pilot ID as a random effect accounting for the repeat testing of pilots. As pretested, the interaction effect between CO_2_ condition and continuous HRV indices was not statistically significant. Therefore, the interaction term was not included in the final GAMM. A logit link function was used to treat examiner ratings as a binomial variable (1: Pass, 0: Fail):

(1) $$y_{i,j,k}=\beta_{1}{+\beta }_{2}*{\mathrm{HRV}+\beta }_{3}*\left({\mathrm{MediumCO}}_{2}\right){+\beta }_{4}*\left({\mathrm{LowCO}}_{2}\right){+\beta }_{5}*\left(\mathrm{Profile}2\right){+\beta }_{6}*\left(\mathrm{Profile}3\right)+\;\beta_{7}*\left(\mathrm{Examiner}2\right){+\beta }_{8}*\left(\mathrm{Examiner}3\right){+\beta }_{9}*\left(\mathrm{DifficultyB}\right){+\beta }_{10}*\left(\mathrm{DifficultyC}\right){+b}_{1i}{+e}_{i,j,k}$$

where *y_i,i,k_* is the passing rate for pilot *i* during profile j on maneuver *k*; *β*_1_ is the fixed intercept; *β*_2_ is the fixed effect of each HRV metric at the maneuver level; *β*_3_ and *β*_4_ are the fixed effects of the medium and low CO_2_ conditions compared to the high CO_2_ condition; *β*_5_ and *β*_6_ are the fixed effects of the second and third sessions compared to the first session; *β*_7_ and *β*_8_ are the fixed effects of Examiners 2 and 3 compared to Examiner 1; *β*_9_ and *β*_10_ are the fixed effects of the Difficulty B and C maneuvers compared to the Difficulty A maneuvers; and *b*_1*i*_ is the random effect of intercept for pilot *i*. Additionally, penalized splines (4 knots, cubic regression) were used to test for the linearity in the relationship between HRV indices and estimates of passing odds. Statistical analyses were performed using the open-source statistical package R version 3.5.0 (R Project for Statistical Computing, Vienna, Austria).

## 3. Results

### 3.1. Influencing Factors of HRV

Summary statistics of the HRV indices by the pilots are presented in Table 2 along with normative short-term HRV data from 44 selected studies involving 21,438 healthy adults at rest conditions (supine or seated), which could be considered as baseline levels for healthy adult population [42]. As shown in Table 2, both the SDNN and RMSSD values of pilots during flight simulations were slightly decreased than the values recorded during the 10-min waiting period before simulations. The LF/HF ratios of pilots were basically consistent before and during flight simulations. The pilots exhibited lower overall HRV (SDNN) and lower parasympathetic regulation (RMSSD) compared with the normative values both before and during the simulations. The ANS activity of pilots tended to be more sympathovagal imbalance, as indicated by higher LF/HF ratios compared with the normative values and the Task Force values (1.5–2.0) [20].

**Figure 2:**
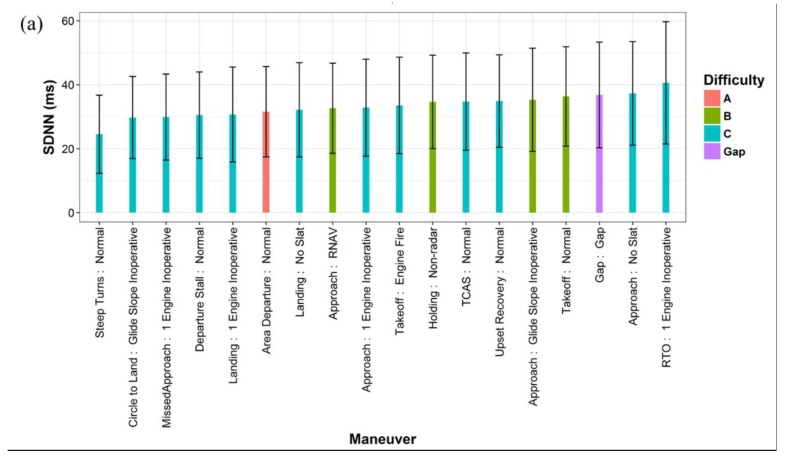

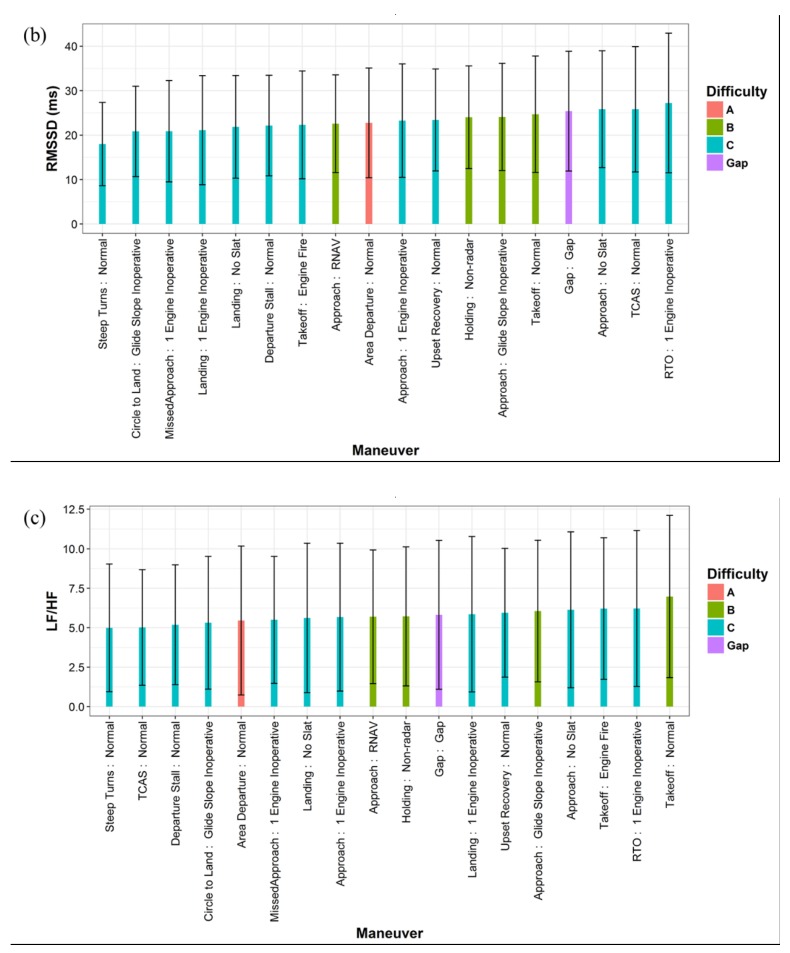
Average HRV values by flight maneuver types and difficulty (the error bars represent the standard deviations): (**a**) SDNN; (**b**) RMSSD; (**c**) LF/HF.

Figure 2 shows the average HRV values on each flight maneuver. As presented in Figure 2a,b, the lowest average SDNN and RMSSD values were found when the pilots conducted the ‘Steep Turns: Normal’ and ‘Circle to Land: Glide Slope Inoperative’ maneuvers, the two most difficult maneuvers with the lowest overall passing rates, 73% and 67%, respectively. In general, the variability was lower on the difficult maneuvers with low passing rates. The higher variability during the ‘Gap’ time may indicate that the pilots could be more relaxed during the cruise periods than conducting active flight maneuvers. As shown in Figure 2c, the average LF/HF ratios were quite close for most maneuvers within the value range of 5 to 7. That means the pilots were dominated by the sympathetic activity when conducting all the maneuvers, reflecting their intensive response to the stressors during the entirety of the flight simulations. The highest LF/HF ratio was found during the three takeoff maneuvers (‘Takeoff: Normal’; ‘RTO: 1 Engine Inoperative’; ‘Takeoff: Engine Fire’), indicating the increased dominance of SNS activity for the pilots during the takeoff phase. In addition, linear mixed effect models were used to test the difference in mean HRV values by active maneuvers compared to the ‘Gap’ time. The results are presented in Table S2.

Table 3 lists the fixed effects of the influencing factors on HRV indices, as estimated by the multivariate linear models. Both the SDNN and RMSSD were higher for the younger pilots relative to the pilots over 50 years old. Meanwhile, the LF/HF ratio was much lower for the pilots of 30 < Age < 40. HRV indices were also related to BMI; the variability was lower, while the LF/HF ratio was higher for the pilots with BMI > 30, which is defined as ‘obesity’ by the Centers for Disease Control and Prevention (CDC). The participants with frequent flight experience as a pilot showed higher variability than those who reported frequently flying in a simulator. However, the LF/HF ratio was slightly higher for the participants with more actual flight experience. No statistically-significant relationship was observed between HRV and CO_2_ condition settings. Exposure to lower CO_2_ concentration had little effect on the variability, but showed a small increase in the LF/HF ratio.

The relationships between the difficulty of maneuver and HRV indices were not consistent, most certainly due to the fact that the majority of maneuvers had a difficulty level of C and the difficulties A and B had a small sample size (Figure 2). Nevertheless, the model results indicated that the SDNN and RMSSD were lower when the pilots conducted the graded maneuvers of any difficulty compared with the ‘Gap’ time.

### 3.2. HRV and Pilot Performance

Table 4, Table 5 and Table 6 lists the GAMM results for the continuous HRV metrics, controlling for other influencing variables. The exponentials of the estimates represent the odds for a pilot passing a maneuver. As shown in the tables, the odds of passing a maneuver slightly increased with the increase of SDNN and RMSSD and decrease of LF/HF ratio. An interquartile range (IQR) increase in SDNN (21.97 ms) and RMSSD (16.00 ms) was associated with an increase of 37% and 22% in the odds of passing a maneuver, respectively. An IQR decrease in LF/HF ratio (4.69) would lead to a 20% increase in the passing odds. In addition, as reported in our previous paper [37], dose-response effects were also observed for the difficulty of maneuver and CO_2_ condition: the odds ratios of passing a maneuver were higher at lower CO_2_ concentration and lower difficulty level of maneuver.

## 4. Discussion

Active airline pilots were flying the simulator under high stress levels as indicated by their lower variability and higher LF/HF ratio, compared with the normative values of healthy adults [42]. The stress of the pilots was generally higher when performing maneuvers during the takeoff, approach and landing phases. Their stress was reduced during the ‘Gap’ periods, which may represent the cruise phase when the pilots are not performing active maneuvers. Lower HRV was associated with aging, high BMI and performing hard maneuvers with low passing rates. Overall, the pilots performed better on maneuvers as rated by the examiners during the flight simulations when their stress was lower, as indicated by the increase of SDNN and RMSSD and decrease of LF/HF ratio, controlling for CO_2_ condition and flight maneuver difficulty.

The findings on the impact of age and BMI on HRV are consistent with other studies. Previous studies of short-term HRV [43,44,45,46] have suggested inverse relationships between age and the time-domain HRV indices. The LF/HF ratio tends to increase with age in population of age <44 years [43], but possibly decrease with aging for elderly subjects of age >44 years [47] or >65 years [48]. Respiratory sinus arrhythmia is a normal physiologic process that becomes less prominent as people age, partly because of decreases in baroreflex sensitivity. This may account for some of the changes in HRV observed in aging populations [49]. The ANS activity is also related to the body weight regulation [50]. In a study of 25 healthy adults [51], increasing BMI is correlated to increased sympathetic activity (higher LF power) and lower parasympathetic activity (lower HF power). It could be speculated that obesity yields to increased energy expenditure as modulated by sympathetic activity. A study of 786 young men [52] also showed that increased BMI was associated with a shift in sympathovagal balance trending towards sympathetic dominance in young adults.

In this study, the relationship between CO_2_ condition and HRV was not significant, which was probably caused by concentrations in the flight simulator at or below 2500 ppm, a level not associated with HRV impacts in prior studies. For example, Kaye et al. [53] investigated the impact of acute CO_2_ exposure on cardiovascular and psychological responses to stress in healthy adults with concentrations from 5% to 35%. They concluded that a single breath of 35% CO_2_ could produce sympathetic and HPA axis activation, indicating the anxiogenic response to hypercapnia by the tested subjects. Lower doses of CO_2_ exposures did not show any significant effects on cardiovascular parameters. Elevating the end-tidal CO_2_ from 5% to 6% could increase HF and LF components of HRV in awake volunteers under both spontaneous and mechanical ventilation [54]. A recent study of indoor air quality and cardiovascular health [55] indicated that no association was observed between HRV and CO_2_ concentration in homes. The CO_2_ concentrations in airplane cabins are much lower than the effect levels [53,54]. For this reason, the ANS activities were not likely on the causal pathway between CO_2_ and cognition of pilots, yet HRV has an independent relationship with the odds of passing a maneuver.

The interaction between stress and pilot performance on different maneuvers may have two aspects. On the one hand, exposure to stress may be detrimental in performing executive-function tasks. Recent studies have signified the relationships between HRV and cognitive function [56]. Reduced cognitive performance associated with lower HRV may be a consequence of the failure of the ANS to properly regulate brain perfusion [57]. More importantly, vagally-mediated HRV has been related to the prefrontal cortex functioning, which is involved in the inhibition of SNS activation [58,59]. Attenuated SNS activity and increased PNS activity are associated with higher prefrontal cortex activity level [58]. Prefrontal cortex activity is correlated with many important cognitive functions such as working memory, sustained attention, behavioral inhibition and general mental flexibility [56,60,61]. All of these cognitive functions are essential for human executive functions that have to do with plan, direct action and self-regulation to perform goal-directed behavior. As a consequence, HRV is also related to cognitive performance of executive tasks. Hansen et al. [62] reported the subjects with higher RMSSD performed better on executive function such as working memory and attention tasks. The following study showed that physically-trained subjects had higher HF component and better cognitive performance on executive tasks than de-trained subjects who did no physical activity for a four week period [63]. A cross-sectional study of 4763 elder participants [64] showed that reduced total variability was associated with poorer cognitive performance as indicated by lower Montreal cognitive assessment (MOCA) score. ZAl Hazzouri et al. [22] collected the short-term ECG data of 2118 middle-age participants and correlated the HRV metrics with their cognitive test performance five years later with a prospective study design. They concluded that higher quartile of SDNN was associated with better executive function as indicated by higher Stroop test score. A study using visuospatial working memory (VSWM) test also showed that the decrement in HRV would lead to poor cognitive performance with an increase in memory load [65]. All of this evidence suggests that stress could profoundly impair goal-directed behavior with increased HPA-axis and SNS activity [66,67], and may contribute to the cognitive deficits observed in mental disorders and extreme environments [56]. As such, we can infer that stress could be detrimental to pilot performance on the hard maneuvers composed of challenging executive tasks being conducted under stressful conditions.

On the other hand, stress could be associated with improved cognitive performance on non-executive function tasks. Exposure to stress may have a positive effect on non-executive function that is driven reflexively by stimulation. Luft et al. [68] studied the differences in athletes’ HRV between executive tasks and non-executive tasks. They found that lower time-domain HRV measures, which means higher stress, was related to faster reaction time on non-executive tasks. The subjects with lower HRV showed faster mean reaction time on a non-executive or easy task under the threat-of-shock condition in which participants were threatened to receive an uncomfortable, but not painful, electric shock through the hand [69]. Stress is thought to be able to enhance memory formation but to impair memory retrieval [70]. Stress can facilitate the processing of sensory information caused by an increase in attention mediated by cortical arousal [71]. Acute exposure to stress may be beneficial to the instructed stimulus-response learning with moderate working memory demand [72]. As such, in some cases, necessary hyper vigilance or so-called eustress possibly make the pilots more alert, enhancing their reaction and cognitive adaptation to maneuvers. For example, the pilots performed well on the ‘Takeoff: Normal’ maneuver, though they had high LF/HF ratios.

In this study, the LF/HF ratio was used as a marker of sympathovagal balance. However, the interpretation of LF remains actively debated, which is considered by some researchers as a measure of sympathetic regulation [73] and by others as a parameter of both sympathetic and vagal regulation [74]. Another interpretation is LF can serve as a marker of sympathetic modulation in some contexts, and more represent parasympathetic activity in other contexts [74,75]. We further analyzed the influencing factors of the LF power and HF power of HRV, as shown in Figure S2 and Table S3. The results show that the changes in LF and HF power were basically in the same direction but with different magnitude. As the GAMM results, an interquartile range (IQR) increase in LF (944 ms^2^) and HF (266 ms^2^) was associated with an increase of 17% and 23% in the odds of passing a maneuver, respectively. The above results indicate that the HF component is highly correlated with the RMSSD measures, both as markers of parasympathetic regulation of heart; the LF component is likely to be influenced by both sympathetic and parasympathetic activity. Consequently, the LF/HF ratio could reflect sympathovagal balance to some extent, but the interpretation of LF and LF/HF ratio still warrants further elucidation.

There are several limitations to consider when interpreting the results of this study. While we had a baseline measurement of HRV before the simulation, the measurements were likely impacted by the stress of the impending simulations. Therefore, the HRV values of pilots may not be directly comparable to the normative HRV values derived from a literature review [42]. A number of studies have revealed large inter-personal variation for the majority of HRV measures [19,42]. The underlying factors for the discrepant values mainly include demographic of subjects, breathing protocols and spectral power analysis methods. In addition, the stress level and performance of pilots presented in this study could be different from those on an actual flight. These simulated flights were not conducted under an actual ‘check ride’ or actual ‘in-flight emergency’. In both of these cases where the pilot’s license and job, and possibly life, is on the line while performing these maneuvers, their stress level could be even higher than what we have demonstrated in this study. Though complying with the PTS standards, the examined maneuvers, such as one engine inoperative and glide slope inoperative, are generally much more challenging than the maneuvers occurred on normally functioning airplanes. As we were interested in the impact on pilot performance, we had the pilots controlling the simulator manually without any auto-pilot aid. Under these conditions, the pilots may more prone to error when conducting difficult maneuvers than during normal flight operations. Our research findings were solely based on male pilots. This reflects the current distribution of pilots in the workforce (94% male), but limit the generalizability to female pilots.

The HRV data that we have collected from airline pilots reflected their physiologic response produced by stressful situations in flight. This is a rare opportunity to evaluate the real-time stress response of pilots in an FAA-approved flight simulator under varying environmental conditions. Studying HRV data in this way can help us better understand how active pilots respond to stress physiologically. The implications of occupational stress and physiologic response can be applied to other workers in high-stress occupations.

## 5. Conclusions

In this study, we studied the HRV of thirty active commercial airline pilots and their performance on flight maneuvers when flying three flight simulation sessions in an FAA-certified A320 flight simulator. The pilots were stressed both before and during the flight simulations, and have higher stress response when conducting advanced flight maneuvers. Lower HRV was associated with age, high BMI and performing difficult maneuvers. The model results showed that exposure to stress could affect pilot performance, independent from the effects of CO_2_ exposure; higher HRV and more balanced ANS activity of a pilot were associated with higher odds of passing a maneuver.

## Figures and Tables

**Table 1:** Basic information of participating pilots.

Category		Percentage
Gender	Male	100%
	Female	0%
Age	30–40	37%
	41–50	27%
	>50	36%
BMI (kg/m^2^)	20–25	40%
	25–30	37%
	>30	23%
Ethnicity	White/Caucasian	20%
	Latino	74%
	Black or African American	3%
	Multiracial	3%
Flight experience	Regularly fly 65+ hours/month as a pilot	70%
	Regularly fly 65+ hours/month in simulation	30%

**Table 2:** Summary statistics of the HRV indices of pilots, compared with the normative values for short-term HRV of healthy adults [42].

HRV Index	Category	Mean	SD	Median	Min	Max
SDNN (ms)	Pilots (during simulations)	34.1	12.7	32.1	12.3	59.3
	Pilots (before simulations)	40.0	11.7	40.9	19.3	58.0
	Normative values	50.0	16.0	51.0	32.0	93.0
RMSSD (ms)	Pilots (during simulations)	23.8	10.2	22.6	6.2	48.0
	Pilots (before simulations)	26.7	9.4	26.7	12.8	48.4
	Normative values	42.0	15.0	42.0	19.0	75.0
LF/HF	Pilots (during simulations)	5.7	2.8	5.5	1.5	14.1
	Pilots (before simulations)	5.5	2.4	5.8	2.8	12.0
	Normative values	2.8	2.6	2.1	1.1	11.6

**Table 3:** The fixed effect estimates on HRV indices, controlling for examiner and flight profile number.

Variable	SDNN (ms) (R-Squared = 0.393)			RMSSD (ms) (R-Squared = 0.443)			LF/HF (R-Squared = 0.189)		
	Estimate	Std. Error	*p*-Value	Estimate	Std. Error	*p*-Value	Estimate	Std. Error	*p*-Value
Intercept	19.21	1.54	<0.001	12.89	1.20	<0.001	5.96	0.49	<0.001
Age > 50	0.00 (Reference)								
41 < Age < 50	11.62	0.86	<0.001	4.13	0.68	<0.001	1.33	0.27	<0.001
30 < Age < 40	18.11	0.78	<0.001	16.56	0.61	<0.001	−3.28	0.25	<0.001
BMI > 30	0.00 (Reference)								
25 < BMI < 30	4.20	0.91	<0.001	5.18	0.71	<0.001	−0.65	0.29	0.026
20 < BMI < 25	3.54	0.85	<0.001	4.37	0.67	<0.001	−0.28	0.27	0.305
Regularly fly 65+ hours/month in simulation	0.00 (Reference)								
Regularly fly 65+ hours /month as a pilot	6.16	0.82	<0.001	0.43	0.64	0.507	1.36	0.26	<0.001
High CO_2_	0.00 (Reference)								
Medium CO_2_	0.64	0.73	0.384	−0.15	0.57	0.797	0.64	0.23	0.006
Low CO_2_	0.10	0.75	0.895	−0.78	0.59	0.182	0.78	0.24	0.001
Gap time	0.00 (Reference)								
Difficulty A	−5.53	1.74	0.002	−2.86	1.36	0.036	−0.36	0.55	0.509
Difficulty B	−2.03	1.37	0.138	−1.40	1.07	0.191	0.22	0.43	0.605
Difficulty C	−4.13	1.27	0.001	−2.66	1.00	0.008	−0.24	0.40	0.558

**Table 4:** GAMM results of SDNN, CO_2_ condition and maneuver difficulty on passing a maneuver, controlling for examiner and flight profile number and treating pilot ID as a random effect.

Variable	Estimate	Odds Ratio (95% CI)	*p*-Value
Intercept	2.02	--	0.004
SDNN	0.014	1.37 (0.93, 2.02) ^1^	0.111
High CO_2_	1.00 (Reference)		
Medium CO_2_	0.50	1.65 (1.06, 2.59)	0.028
Low CO_2_	0.63	1.87 (1.17, 3.01)	0.009
Difficulty A	1.00 (Reference)		
Difficulty B	−0.46	0.63 (0.17, 2.27)	0.478
Difficulty C	−1.43	0.24 (0.07, 0.79)	0.020

^1^ Odds ratio for an IQR increase in SDNN (21.97 ms).

**Table 5:** GAMM results of RMSSD, CO_2_ condition and maneuver difficulty on passing a maneuver, controlling for examiner and flight profile number and treating pilot ID as a random effect.

Variable	Estimate	Odds Ratio (95% CI)	*p*-Value
Intercept	2.19	--	0.001
RMSSD	0.013	1.22 (0.87, 1.73) ^1^	0.251
High CO_2_	1.00 (Reference)		
Medium CO_2_	0.52	1.68 (1.07, 2.63)	0.024
Low CO_2_	0.63	1.88 (1.17, 3.02)	0.009
Difficulty A	1.00 (Reference)		
Difficulty B	−0.45	0.64 (0.18, 2.30)	0.478
Difficulty C	−1.44	0.24 (0.07, 0.79)	0.020

^1^ Odds ratio for an IQR increase in SDNN (16.00 ms).

**Table 6:** GAMM results of LF/HF, CO_2_ condition and maneuver difficulty on passing a maneuver, controlling for examiner and flight profile number and treating pilot ID as a random effect.

Variable	Estimate	Odds Ratio (95% CI)	*p*-Value
Intercept	2.67	--	<0.001
LF/HF	−0.038	1.20 (0.94, 1.51) ^1^	0.137
High CO_2_	1.00 (Reference)		
Medium CO_2_	0.56	1.76 (1.12, 2.75)	0.014
Low CO_2_	0.65	1.92 (1.19, 3.08)	0.007
Difficulty A	1.00 (Reference)		
Difficulty B	−0.44	0.64 (0.18, 2.31)	0.497
Difficulty C	−1.48	0.23 (0.07, 0.76)	0.016

^1^ Odds ratio for an IQR decrease in LF/HF (4.69).

## Supplementary Materials

The following are available online at http://www.mdpi.com/1660-4601/16/2/237/s1. Figure S1: Overall passing rates of pilots on each maneuver in the flight simulator (the error bars represent the standard errors), Figure S2: Average LF and HF power of HRV by flight maneuver types and difficulty, Table S1: Flight maneuvers and performance criteria, Table S2: Difference in mean HRV values by flight maneuver types (referent: Gap time), treating pilot as a random intercept, Table S3: The fixed effect estimates on LF and HF, controlling for examiner and flight profile number.
