# Supplementary material for: Heart Rate Variability and Performance of Commercial Airline Pilots during Flight Simulations
Source: Int J Environ Res Public Health. 2019 Jan 16;16(2):237. doi: 10.3390/ijerph16020237 (PMC6352143; doi:10.3390/ijerph16020237)
Supplement: Supplementary file 1 [file ijerph-16-00237-s001.pdf]

Supplemental material

Heart rate variability and performance of commercial airline pilots during flight simulations

Xiaodong Cao[1], Piers MacNaughton[1], Leslie R. Cadet[1], Jose Guillermo Cedeno-Laurent[1], Skye Flanigan[1], Jose Vallarino[1], Deborah Donnelly-McLay[1], David C. Christiani[1,2], John D. Spengler[1], Joseph G. Allen[1,*]

Affiliation
[1] Department of Environmental Health, Harvard T.H. Chan School of Public Health, Boston, MA, USA.
[2] Department of Medicine, Massachusetts General Hospital/Harvard Medical School, Boston, MA, USA.

Corresponding Author:
Joseph G. Allen, DSc, MPH
Assistant Professor
Harvard T.H. Chan School of Public Health
401 Park Drive
Landmark Center, 404-L
Boston, MA 02215
e: JGAllen@hsph.harvard.edu
o: 617-384-8475

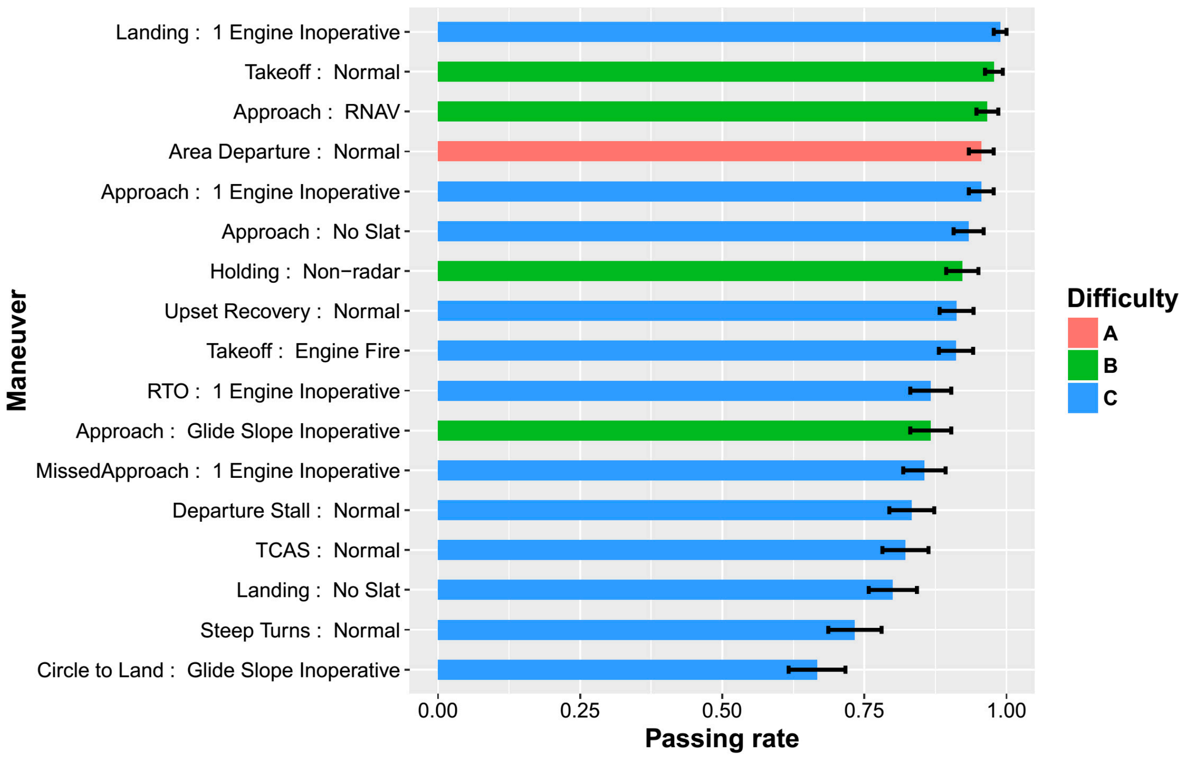

Figure S1. Overall passing rates of pilots on each maneuver in the flight simulator (the error bars represent the standard errors).

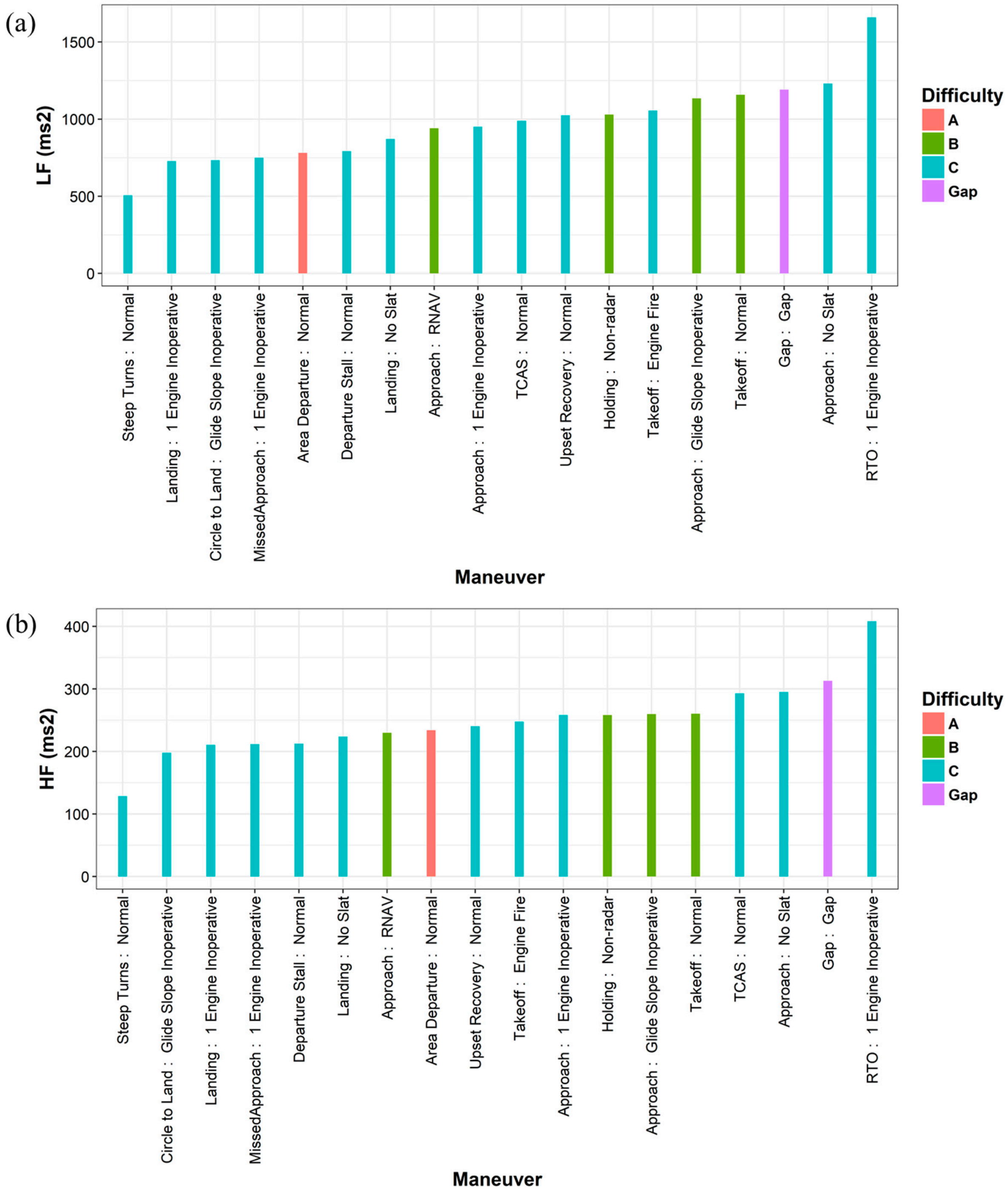

Figure S2. Average LF and HF power of HRV by flight maneuver types and difficulty.

Table S1. Flight maneuvers and performance criteria.

Maneuver	Type	Maneuver Order Boston	NYC	DC	Difficulty	Performance Metric	Description
Takeoff	Normal	1	3	2	B	1. Straight Heading during takeoff	Absolute deviation of bank angle from 0 degrees during maneuver
Area Departure					A	2. Constant Pitch during Ascent	Absolute deviation of pitch attitude from 15 degrees from 400-1500ft
Traffic Collision Avoidance (TCAS)					C	3. Altitude Loss during TCAS	Change in altitude from beginning of TCAS to minimum/maximum altitude.
Approach	No Slat/Flap Malfunction				C	4. Constant Altitude at Waypoint	Absolute deviation of altitude from 1700ft at waypoint
Landing					C	5. Straight Heading during Landing	Absolute deviation of bank angle from 0 degrees below 400ft of altitude
Rejected Takeoff (RTO)	1 Engine Inoperative	2	1	3	C	6. Quick Deceleration after Malfunction	Time (s) to complete stop after beginning deceleration
Steep Turns	Normal				C	7. Constant Bank during Steep Turns	Absolute deviation of roll rate from 0 degrees/s during steep turns
Departure Stall					C	8. Smooth Recovery after Stall	R^2 for a linear regression of the rate of airspeed increase after stall until return to initial airspeed
Upset Recovery					C	9. Smooth Recovery after Upset	R^2 for a linear regression of altitude change after upset until return to 50% of initial altitude
Holding	Non-radar				B	10. Constant Altitude during Holding	Absolute deviation of altitude from 1500ft during holding
Approach	Glide Slope Inoperative				B	11. Constant Altitude at Waypoint	Absolute deviation of altitude from 1500ft at waypoint
Circle to Land					C	12. Constant Altitude during Circle to Land	Absolute deviation of altitude from 700ft during maneuver
Takeoff	Engine Fire	3	2	1	C	13. Straight Heading during takeoff	Absolute deviation of bank angle from 0 degrees
Approach	RNAV				B	14. Straight Heading during Maneuver	Absolute deviation of bank angle from 0 degrees
Missed Approach	1 Engine Inoperative				C	15. Altitude at missed approach	Minimum altitude before rejecting landing
Approach					C	16. Constant Altitude at Waypoint	Absolute deviation of altitude from 1700ft at waypoint
Landing					C	17. Straight Heading during Landing	Absolute deviation of bank angle from 0 degrees below 400ft of altitude

Table S2. Difference in mean HRV values by flight maneuver types (referent: Gap time), treating pilot as a random intercept.

	SDNN			RMSSD			LF/HF		
	Estimate	Std.Error	p-Value	Estimate	Std.Error	p-Value	Estimate	Std.Error	p-Value
Intercept	36.04	2.33	<0.001	24.93	1.86	<0.001	5.82	0.52	<0.001
Gap time					Reference				
Approach: 1 Engine Inoperative	-4.10	0.22	<0.001	-2.45	0.17	<0.001	-0.06	0.08	0.489
Approach: Glide Slope Inoperative	-1.35	0.21	<0.001	-0.98	0.17	<0.001	0.12	0.08	0.148
Approach: No Slat	0.20	0.22	0.375	0.25	0.17	0.150	0.33	0.08	<0.001
Approach: RNAV	-3.86	0.22	<0.001	-2.52	0.17	<0.001	-0.16	0.08	0.055
Area Departure: Normal	-5.52	0.36	<0.001	-2.91	0.29	<0.001	-0.31	0.14	0.029
Circle to Land: Glide Slope Inoperative	-7.47	0.32	<0.001	-4.92	0.25	<0.001	-0.48	0.12	<0.001
Departure Stall: Normal	-6.05	0.24	<0.001	-3.14	0.19	<0.001	-0.66	0.09	<0.001
Holding: Non-radar	-2.10	0.23	<0.001	-1.19	0.19	<0.001	-0.17	0.09	0.060
Landing: 1 Engine Inoperative	-6.50	0.52	<0.001	-4.64	0.41	<0.001	-0.03	0.20	0.882
Landing: No Slat	-4.92	0.36	<0.001	-3.57	0.29	<0.001	-0.32	0.14	0.021
Missed Approach: 1 Engine Inoperative	-7.20	0.52	<0.001	-4.69	0.41	<0.001	-0.37	0.20	0.068
RTO: 1 Engine Inoperative	4.56	0.62	<0.001	2.69	0.49	<0.001	0.17	0.24	0.472
Steep Turns: Normal	-11.88	0.31	<0.001	-7.10	0.25	<0.001	-0.86	0.12	<0.001
Takeoff: Engine Fire	-3.19	0.50	<0.001	-2.91	0.40	<0.001	0.31	0.19	0.105
Takeoff: Normal	-0.19	0.55	0.734	-0.46	0.44	0.296	1.10	0.21	<0.001
TCAS: Normal	-1.87	0.35	<0.001	0.25	0.28	0.371	-0.56	0.14	<0.001
Upset Recovery: Normal	-1.22	0.34	<0.001	-1.53	0.27	<0.001	0.13	0.13	0.312

Table S3. The fixed effect estimates on LF and HF, controlling for examiner and flight profile number.

Variable	LF (ms^2) (R-squared = 0.258)			HF (ms^2) (R-squared = 0.332)		
	Estimate	Std. Error	p-Value	Estimate	Std. Error	p-Value
Intercept	191.72	111.10	0.085	86.75	32.77	0.008
Age > 50			0.00 (Reference)			
41 < Age < 50	827.46	62.54	< 0.001	105.86	18.44	< 0.001
30 < Age < 40	885.57	56.61	< 0.001	381.59	16.70	< 0.001
BMI > 30			0.00 (Reference)			
25 < BMI < 30	202.04	66.02	0.002	75.66	19.47	< 0.001
20 < BMI < 25	10.75	61.56	0.861	36.01	18.16	0.048
Regularly fly 65+ hours/month in simulation			0.00 (Reference)			
Regularly fly 65+ hours /month as a pilot	507.14	59.28	< 0.001	22.38	17.48	0.201
High CO_2			0.00 (Reference)			
Medium CO_2	20.55	52.96	0.698	-23.47	15.62	0.133
Low CO_2	33.87	54.25	0.533	-49.53	16.00	0.002
Gap time			0.00 (Reference)			
Difficulty A	-435.25	126.11	0.001	-80.91	37.19	0.030
Difficulty B	-139.25	99.10	0.160	-53.21	29.23	0.069
Difficulty C	-271.24	92.21	0.003	-65.13	27.20	0.017
